# Group B Streptococcal Endocarditis in Obstetric and Gynecologic Practice

**DOI:** 10.1080/10647440300025507

**Published:** 2003

**Authors:** Antonio Crespo, Avi S. Retter, Bennett Lorber

**Affiliations:** Section of Infectious Diseases Department of Medicine Temple University School of Medicine and Hospital Broad and Ontario Streets Philadelphia PA 19140 USA

## Abstract

*Background:* We describe a case and review ten other instances of group B streptococcal endocarditis in the setting of obstetric and gynecologic practice reported since the last review in 1985.

*Case:* Abortion remains a common antecedent event, but in contrast to earlier reports, most patients did not have underlying valvular disease, the tricuspid valve was most often involved, and mortality was low. Patients with tricuspid valve infection tended to have a subacute course, whereas those with aortic or mitral involvement typically had a more acute, fulminant course.

*Conclusion:* Despite an improvement in mortality, morbidity remains high, with 8 of 11 patients having clinically significant emboli.


Infect Dis Obstet Gynecol 2003;11:109–115

### Group B streptococcal endocarditis in obstetric and gynecologic practice

**Antonio Crespo, Avi S. Retter and Bennett Lorber**

*Section of Infectious Diseases, Department of Medicine, Temple University School of Medicine and Hospital, Philadelphia, PA*

***Background:*** We describe a case and review ten other instances of group B streptococcal endocarditis in the setting of obstetric and gynecologic practice reported since the last review in 1985.
***Case:*** Abortion remains a common antecedent event, but in contrast to earlier reports, most patients did not have underlying valvular disease, the tricuspid valve was most often involved, and mortality was low. Patients with tricuspid valve infection tended to have a subacute course, whereas those with aortic or mitral involvement typically had a more acute, fulminant course.
***Conclusion:*** Despite an improvement in mortality, morbidity remains high, with 8 of 11 patients having clinically significant emboli.

Key words: ENDOCARDITIS; GROUP B STREPTOCOCCUS; *STREPTOCOCCUS AGALACTIAE*; OBSTETRICAL COMPLICATION; INFECTION

*Streptococcus agalactiae*, better known as group B streptococcus (GBS), is a common cause of neonatal sepsis and meningitis. The organism is acquired by the neonate from the vaginal flora of the mother at the time of parturition. In adults, the epidemiology of GBS infections has changed from an uncommon cause of puerperal sepsis in the pre-antibiotic era to that of an infrequent, but important etiology of bacteremia and locally invasive infections in diabetics, alcoholics and others with chronic debilitating diseases[1–4]. Although GBS is an uncommon cause of endocarditis when all etiologies are considered, endocarditis accounts for 10% of reported invasive infections due to this organism[4–7].

We here report a case of GBS prosthetic valve endocarditis associated with an obstetrical procedure, and we review cases of GBS endocarditis in obstetric and gynecologic practice reported in the English-language literature since 1985, the time of the last review of this subject[8].

### METHODS

A computerized, English-language literature search via the Medline database was performed for reported cases of infective endocarditis caused by GBS that were associated with obstetric or gynecologic conditions and/or procedures. The search was restricted to the period between January 1985 and June 2002. The following key words were used in the search: ‘endocarditis’, ‘group B streptococcus’, ‘*Streptococcus agalactiae*’, ‘pregnancy’, ‘abortion’, ‘obstetric’ and ‘gynecology’.

Correspondence to: Bennett Lorber, MD, Section of Infectious Diseases, Temple University Hospital, Broad and Ontario Streets, Philadelphia, PA 19140, USA. Email: lorberb@tuhs.temple.edu

© 2003 The Parthenon Publishing Group

### CASE REPORT

A 37-year-old woman with a history of infective endocarditis 11 years earlier that necessitated replacement of both aortic and mitral valves came to the emergency department with complaints of fever, chills, shortness of breath and left-sided pleuritic pain of 4 days' duration. Six months before her acute illness, while she was 8 weeks pregnant, she was hospitalized for a near syncopal episode and was found to have critical aortic stenosis. An uneventful elective abortion was performed due to concerns about further hemodynamic complications during pregnancy. The patient received 2 g of ampicillin and 100 mg of gentamicin intravenously prior to the dilation and curettage, followed by 1 g of ampicillin intravenously 6 hours later. Surgery to repair the aortic stenosis was recommended, but the patient refused this. Approximately 2 months after the abortion, she presented to the gynecology clinic complaining of heavy vaginal bleeding and pelvic pain. The physical examination was unremarkable and a Papanicolaou cervical smear was performed. The patient received a course of oral medroxyprogesterone and had initial resolution of menorrhagia. Approximately 1 month prior to her admission she presented again to her gynecologist with a 2-week history of recurrent vaginal bleeding and pelvic pain. Another course of medroxyprogesterone was prescribed.

The patient had a history of intravenous drug use, but had quit 18 months prior to her presentation. She had snorted cocaine 2 days before her admission. Apart from the elective abortion she denied any surgical procedure or dental work during the months preceding her illness.

The physical examination that was performed on admission revealed a well-developed, pale woman who was very anxious about her illness. Her temperature was 39.7°C; heart rate was 120 beats/minute; blood pressure was 100/66 mmHg and respiration rate was 24 breaths/minute. A 3/6 early systolic ejection murmur was heard at the base of the heart radiating to the apex. No stigmata of infective endocarditis were present. A chest X-ray revealed no abnormalities, and the electrocardiogram showed sinus tachycardia. The patient's white blood cell count was 5600 cells/mm$^3$ with 80% neutrophils, 2% bands, 7% lymphocytes and 11% monocytes. She was not anemic. Her serum sodium concentration was 127 mmol/l (normal range 135–145 mmol/l), potassium concentration was 2.9 mmol/l (normal range 3.5–5.0 mmol/l) and phosphorus concentration was 2.2 mg/dl (normal range 2.5–4.5 mg/dl). A urine drug screen was negative.

The patient was given vancomycin, gentamicin and rifampin for suspected infective endocarditis. Two blood cultures from the day of admission grew *Streptococcus agalactiae* (group B streptococcus), and the antibiotic regimen was changed to ampicillin 2 g every 6 hours and gentamicin 100 mg every 8 hours, both administered intravenously. A transthoracic echocardiogram revealed a vegetation on the outflow side of the prosthetic aortic valve (Figure 1) and a possible vegetation on the left ventricular side of the prosthetic mitral valve. The area of the aortic valve was 0.8 cm$^2$.

During the hospital course, the patient developed worsening of her left lower chest pleuritic pain and a new finding of tender splenomegaly. Tender nodules on her fingers consistent with Osler's nodes were also found, and she complained of pain in the anterior aspect of

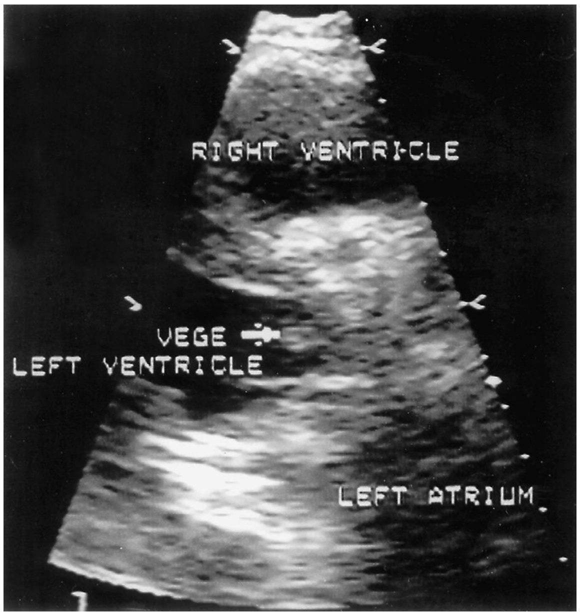

**Figure 1** Transthoracic echocardiogram from a patient with group B streptococcal endocarditis demonstrating a vegetation on the outflow side of her prosthetic aortic valve

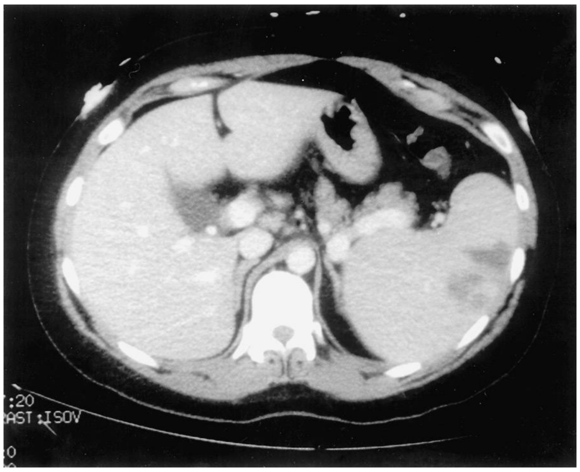

**Figure 2** Computed tomography image of the abdomen from a patient with group B streptococcal endocarditis demonstrating multiple, hypodense, wedge-shaped densities in the spleen consistent with septic emboli both lower extremities. Follow-up chest X-rays showed left lower lobe atelectasis with a small pleural effusion, and computed tomography showed multiple hypodense, peripheral, wedge-shaped areas in the spleen consistent with septic emboli (Figure 2). Subsequent blood cultures throughout her hospitalization showed no growth, and after 10 days of antimicrobial treatment she became afebrile.

Initially, replacement of the aortic and mitral valves was planned, but the patient refused to undergo surgery. It was decided to follow her clinical course closely and intervene if signs of heart failure developed. A transesophageal echocardiogram taken 3 weeks after admission showed no apparent vegetations and an improved aortic valve area of 1.1 $cm^2$. The patient's clinical condition improved, and she completed 6 weeks of antimicrobial therapy. She was last seen 10 months after hospitalization, and has remained asymptomatic.

### DISCUSSION

In women of childbearing age, colonization of the genital tract with GBS is common and serves as a reservoir for a variety of infections, including maternal endometritis and bacteremia as well as neonatal sepsis and meningitis[1]. Despite this, infective endocarditis in obstetric and gynecologic practice is rare. Seaworth and Durack[7] reviewed 99 cases of all-cause endocarditis in the obstetrical and gynecological literature from 1940 to 1983. There were only seven cases caused by GBS, but GBS accounted for 16% of the cases following abortion.

Pregnancy-associated GBS endocarditis was reviewed in 1985 by Sexton *et al.*[8], who reported two new cases and collected 19 more cases from the literature, including cases from both the pre- and post-antibiotic era. Most of the patients had pre-existing valvular heart disease. Prior to the advent of antimicrobial therapy, almost all cases involved the mitral valve, but in the post-antibiotic era the tricuspid and aortic valves were involved as often as the mitral valve. An acute course, similar to that seen in staphylococcal endocarditis and in contrast to the more subacute course of *viridans* streptococcal disease, was the norm. A high rate of complications was also observed – both index cases developed annular abscesses and had a fatal outcome. A decrease in mortality from 67% to 43% was observed after the advent of antibiotics.

Including our report, 11 cases of GBS endocarditis have been published in the English-language literature since 1985 (Table 1). The majority of patients (64%) did not have a predisposing valvular condition. The exceptions were one instance of mitral valve prolapse with regurgitation[10], one patient with rheumatic mitral valve disease[17] and two patients with mechanical valves[17], one of whom is our patient who underwent bivalvular replacement surgery several years prior to her presentation. Since 1985, in contrast to earlier reports, the tricuspid valve was the most commonly involved valve (6 of 11 cases), the course was subacute rather than fulminant, and valve replacement was required in only four patients. In patients with non-tricuspid valve involvement, the clinical course was typically more aggressive, resembling that of staphylococcal endocarditis. What is even more interesting is that only one patient died (mortality rate of 9%), a dramatic decrease from the mortality rate of around 40–70% noted in earlier series[2,6,8,18]. A low mortality rate (12.9%) was also found in a review of all β-hemolytic streptococcal endocarditis; GBS was the most common isolate. A recently published review[17] of 30 cases of GBS

**Table 1** Cases of group B streptococcal endocarditis in obstetrics and gynecology since 1985

| *Case number* | *Age (years)* | *Obstetric/gynecological condition or procedure involved* | *Underlying heart valve disease* | *Valve* | *Duration of symptoms before diagnosis* | *Heart valve replaced* | *Outcome* | *Complications* | *Therapy* | *Year and reference* |
|---|---|---|---|---|---|---|---|---|---|---|
| 1 | 18 | Premature rupture of membranes | None | Aortic | 3 days | No | Cured | None | Penicillin G, 20 million units/day for 28 days, plus gentamicin, 180 mg/day for 14 days | 1985[9] |
| 2 | 30 | Vaginal delivery | Mitral valve prolapse with regurgitation | Mitral | 4 weeks | No | Cured | Amaurosis fugax | Penicillin G, 16 million units/day for 45 days, plus gentamicin 240 mg/day for 2 days | 1987[10] |
| 3 | 18 | Elective abortion | None | Tricuspid | Several weeks | No | Cured | Septic pulmonary emboli | Penicillin G, 24 million units/day, plus gentamicin, 180 mg/day for 6 weeks | 1990[11] |
| 4 | 30 | Elective abortion | None | Tricuspid | 4 weeks | Yes | Cured | Septic pulmonary emboli, first-degree heart block | Vancomycin for 28 days | 1991[12] |
| 5 | 36 | Papanicolaou cervical smear | None | Tricuspid | 7 days | Yes | Cured | Severe tricuspid regurgitation | Penicillin plus gentamicin for 3 weeks before TVR followed by ampicillin plus gentamicin for 6 weeks | 1997[13] |
| 6 | 33 | Elective abortion | None | Tricuspid | 4 weeks | Yes | Cured | Septic pulmonary emboli, valve ring abscess | Penicillin plus gentamicin for 6 weeks | 1998[14] |
| 7 | 24 | Elective abortion | None | Tricuspid | 4 weeks | No | Cured | Septic pulmonary emboli, right-sided heart failure | Ampicillin plus gentamicin for 6 weeks | 2000[15] |

*Continued*

**Table 1** *continued*

| | | | | | | | | | | |
|---|---|---|---|---|---|---|---|---|---|---|
| 8 | 60 | Giant pyomyoma | None | Tricuspid | 12 weeks | No | Cured | None | Penicillin G, 18 million units/day, plus amikacin 600 mg/day added on day 11 (unknown duration) | 2001[16] |
| 9 | 42 | Genital infection | Rheumatic valve disease | Mitral | 10 days | No | Cured | None | Penicillin G plus gentamicin | 2002[17] |
| 10 | 32 | Genital infection | Prosthetic mitral valve | Mitral | 5 days | Yes | Died | Stroke | Penicillin G plus gentamicin | 2002[17] |
| 11 | 37 | Elective abortion, Papanicolaou cervical smear, vaginal bleeding | Prosthetic aortic and mitral valves | Aortic and possible mitral valve | 4 days | No | Cured | Left-sided pleurisy, septic splenic emboli, emboli to extremities | Ampicillin 8 g/day, plus gentamicin, 300 mg/day, for 6 weeks | 2002 Present case |

TVR, tricuspid valve replacement endocarditis since 1980 found a similar trend towards lower mortality rate (21%) compared with earlier cases. In this review, all patients with prosthetic valve endocarditis died (five cases). The reason for this markedly improved outcome is not obvious, and it is not explained by changes in antimicrobial therapy or surgical technique. Prompt use of blood cultures in febrile patients and the more frequent use of echocardiography might account for earlier diagnosis, earlier surgical intervention when necessary, and improved survival, but this is only speculation. Other factors that might be associated with a favorable outcome in this group include young age and tricuspid rather than aortic or mitral involvement. Despite the improvement in mortality rate, the morbidity rate remains high, with 8 of 11 patients having clinically significant emboli.

With regard to the obstetric or gynecologic conditions or procedures that preceded the development of endocarditis, abortion continued to be most commonly implicated. A report of one case following a Papanicolaou cervical smear[13] suggests that even simple, minimally invasive procedures can serve to introduce bacteria into the bloodstream. One patient with an infected uterine leiomyoma presented with tricuspid valve endocarditis and had not had any intervention prior to her admission to hospital[16]. The only case following a vaginal delivery was in a woman with known valvular heart disease[10]. None of the patients in this report were active intravenous drug users.

The pathogenesis of infection in the present case is unclear. The patient had a therapeutic abortion 6 months before presenting with endocarditis, but she had received appropriate antimicrobial prophylaxis before the procedure. Recurrent vaginal bleeding and a Papanicolaou cervical smear performed 2 months prior to her admission could have served as sources of GBS. Although no cases with vaginal bleeding as the sole predisposing factor were encountered in our literature search, we believe that our patient's recurrent episodes of vaginal bleeding may well have enabled the organism to invade her bloodstream, thereby leading to infection of her prosthetic heart valves.

As in previous reports, treatment routinely involved the use of a β-lactam antibiotic plus an aminoglycoside for 4 to 6 weeks. The antibiotic of choice for GBS endocarditis has been penicillin or ampicillin[19]. Vancomycin is used in patients with β-lactam allergy. Since the minimum inhibitory concentration of penicillin for GBS is usually four- to eight-fold higher than that for other streptococci[5,20], most authorities recommend the addition of an aminoglycoside as a synergistic agent to high doses of penicillin or ampicillin for at least the first 2 weeks. The optimum duration of treatment has not been established, but intravenous antibiotics are routinely given for 4 to 6 weeks. The role of chemoprophylaxis for endocarditis in individuals with known valvular heart disease outside the intrapartum setting has not been well studied. Recommendations for endocarditis prophylaxis from the American Heart Association[21] should be followed, but individual decisions need to be made when dealing with patients who have high-risk cardiac conditions, as was illustrated by our patient.

### REFERENCES

1. Lerner P, Gopalakrishna KV, Wolinsky E, *et al.* Group B streptococcus *(S. agalactiae)* bacteremia in adults: analysis of 32 cases and review of the literature. *Medicine* 1977;56:457–73
2. Gallagher PG, Watanakunakorn C. Group B streptococcal endocarditis: report of seven cases and review of the literature, 1962–1985. *Rev Infect Dis* 1986;8:175–88
3. Schwartz B, Schuchat A, Oxtoby M, *et al.* Invasive group B streptococcal disease in adults. A population-based study in metropolitan Atlanta. *J Am Med Assoc* 1991;266:1112–14
4. Farley MM, Harvey C, Stull T, *et al.* A population-based assessment of invasive disease due to group B streptococcus in nonpregnant adults. *N Engl J Med* 1993;328:1807–11
5. Bayer A, Chow AW, Anthony BF, *et al.* Serious infections in adults due to group B streptococci. Clinical and serotypic characterization. *Am J Med* 1976;61:498–503

6. Gallagher PG, Watanakunakorn C. Group B streptococcal bacteremia in a community teaching hospital. *Am J Med* 1985;78:795–800
7. Seaworth BJ, Durack DT. Infective endocarditis in the obstetric and gynecologic practice. *Obstet Gynecol* 1986;154:180–8
8. Sexton DJ, Rockson SG, Hempling RE, *et al*. Pregnancy-associated group B streptococcal endocarditis: a report of two fatal cases. *Obstet Gynecol* 1985;66:44–7S
9. Backes RJ, Wilson WR, Geraci JE. Group B streptococcal infective endocarditis. *Arch Intern Med* 1985;145:693–6
10. Strasberg GD. Postpartum group B streptococcal endocarditis associated with mitral valve prolapse. *Obstet Gynecol* 1987;70:485–7
11. Atri ML, Cohen DH. Group B streptococcus endocarditis following second-trimester abortion. *Arch Intern Med* 1990;149:2579–80
12. Vartian CV, Septimus EJ. Tricuspid-valve group B streptococcal endocarditis following elective abortion. *Rev Infect Dis* 1991;13:997–8
13. Mong K, Taylor D, Muzyka T, *et al*. Tricuspid endocarditis following a Papanicolaou smear: case report. *Can J Cardiol* 1997;13:895–6
14. Azzam ZS, Ron Y, Oren I, *et al*. Group B streptococcal tricuspid-valve endocarditis: a case report and review of the literature. *Int J Cardiol* 1998; 64:259–63
15. Kangavari S, Collins J, Cercek B, *et al*. Tricuspid valve group B streptococcal endocarditis after an elective termination of pregnancy. *Clin Cardiol* 2000;23:301–3
16. Genta PR, Dias MLN, Janiszewski TA, *et al*. *Streptococcus agalactiae* endocarditis and giant pyomyoma simulating ovarian cancer. *South Med J* 2001;94:508–11
17. Sambola A, Miro JM, Tornos MP, *et al*. *Streptococcus agalactiae* infective endocarditis: analysis of 30 cases and review of the literature, 1962–1998. *Clin Infect Dis* 2002;34:1576–84
18. Scully BE, Spriggs D, Neu HC. *Streptococcus agalactiae* (Group B) endocarditis: a description of twelve cases and review of the literature. *Infection* 1987;15:169–76
19. Baddour LM. Infective endocarditis caused by β-hemolytic streptococci. *Clin Infect Dis* 1998; 26:66–71
20. Baker CN, Thornsberry C, Facklam RR. Synergism, killing kinetics and antimicrobial susceptibility of group A and B streptococci. *Antimicrob Agents Chemother* 1981;19:716–25
21. Dajani AS, Taubert KA, Wilson W, *et al*. Prevention of bacterial endocarditis. Recommendations by the American Heart Association. *J Am Med Assoc* 1997;277:1794–801

RECEIVED 02/07/03; ACCEPTED 05/06/03
